# Generation of Distal Airway Epithelium from Multipotent Human Foregut Stem Cells

**DOI:** 10.1089/scd.2014.0512

**Published:** 2015-03-10

**Authors:** Nicholas R.F. Hannan, Fotios Sampaziotis, Charis-Patricia Segeritz, Neil A. Hanley, Ludovic Vallier

**Affiliations:** ^1^ Anne McLaren Laboratory for Regenerative Medicine, Department of Surgery, Wellcome Trust-Medical Research Council Stem Cell Institute, University of Cambridge, Cambridge, United Kingdom.; ^2^ Faculty of Medical and Human Sciences, Centre for Endocrinology and Diabetes, Manchester Academic Health Sciences Centre, Institute of Human Development, University of Manchester, Manchester, United Kingdom.; ^3^ Wellcome Trust Sanger Institute, Hinxton, United Kingdom.

## Abstract

Collectively, lung diseases are one of the largest causes of premature death worldwide and represent a major focus in the field of regenerative medicine. Despite significant progress, only few stem cell platforms are currently available for cell-based therapy, disease modeling, and drug screening in the context of pulmonary disorders. Human foregut stem cells (hFSCs) represent an advantageous progenitor cell type that can be used to amplify large quantities of cells for regenerative medicine applications and can be derived from any human pluripotent stem cell line. Here, we further demonstrate the application of hFSCs by generating a near homogeneous population of early pulmonary endoderm cells coexpressing NKX2.1 and FOXP2. These progenitors are then able to form cells that are representative of distal airway epithelium that express NKX2.1, GATA6, and cystic fibrosis transmembrane conductance regulator (CFTR) and secrete SFTPC. This culture system can be applied to hFSCs carrying the CFTR mutation Δf508, enabling the development of an in vitro model for cystic fibrosis. This platform is compatible with drug screening and functional validations of small molecules, which can reverse the phenotype associated with CFTR mutation. This is the first demonstration that multipotent endoderm stem cells can differentiate not only into both liver and pancreatic cells but also into lung endoderm. Furthermore, our study establishes a new approach for the generation of functional lung cells that can be used for disease modeling as well as for drug screening and the study of lung development.

## Introduction

Lung disorders are a leading cause of death worldwide, second only to cardiovascular disease. Multiple cell types participate in the disease pathogenesis, including epithelial cells, myofibroblasts, and cells of the immune system. However, respiratory epithelial cells (RECs) play the most pivotal role in coordinating the complex cellular interactions leading to disease [1]. Indeed, the pulmonary epithelium shows a unique potential for controlling lung repair, remodeling, and fibrosis through epithelial–mesenchymal interactions and orchestrating inflammatory responses through secretion of pro-inflammatory cytokines [1,2]. Furthermore, the role of RECs as the interface between the respiratory tissue and the external environment renders them a prime target for inhaled therapeutic agents [1]. Therefore, the development of robust platforms for studying the respiratory epithelium could advance our insight into the mechanisms underlying pulmonary diseases and contribute toward the generation of novel therapeutic agents. Nevertheless, existing in vivo models are limited by intra-species variability, while a long-term primary RECs culture is complicated by technical challenges and poor access to primary tissue.

The technology of human-induced pluripotent stem cells (hIPSCs) has contributed toward addressing this challenge through the development of protocols for the generation of RECs in vitro [3,4]. However, these existing systems are limited by significant variability in differentiation capacity among lines, as well as by an inability to produce sufficiently pure populations of cell sub-types without genetic manipulation [5]. Ultimately, this limits their overall efficiency and makes the derivation of large cohorts of patient-specific RECs problematic. We recently developed a platform for the generation of human foregut stem cells (hFSCs), with the potential for overcoming such issues [6]. hFSCs resemble foregut progenitor cells from which multiple endodermal organs originate, including the liver, pancreas, thyroid, thymus, and lungs [7]. These multipotent stem cells can be derived from any human embryonic stem cell (hESC) or hIPSC with a high efficiency. Furthermore, they can self-renew in vitro for a prolonged period of time and maintain their capacity to differentiate into endoderm lineages, such as the liver and pancreatic cells [6]. However, differentiation of hFSCs into RECs that could overcome issues associated with poor efficiency and variability between lines has not yet been demonstrated.

In the mouse, foregut specification into lung bud is marked by the expression of the transcription factors NKX2.1 and FOXP2 [8], while thyroid progenitors also express NKX2.1 but in combination with PAX8 and HHEX [9,10]. Airway epithelium differentiates from NKX2.1/FOXP2 progenitors into secretory Clara cells, neuroendocrine cells, mucus-producing goblet cells, and ciliated cells (See Table 1 for summary of lineage markers used for this study). At the distal tip of the lung, two types of cells are also produced: the alveolar epithelial cells (AECs) Type I (AECTI) and Type II (AECTII). In the mature lung, AECTI cells are responsible for gas exchange and express a variety of markers such as NKX2.1, GATA6, and the cystic fibrosis transmembrane conductance regulator (CFTR) [11,12]. The AECTII, which itself gives rise to the AECTI, also expresses NKX2.1, GATA6, and CFTR as well as the surfactant proteins that reduce the alveolar surface tension. While the mature distal lung epithelium expresses some markers in common with the embryonic lung bud (NKX2.1, GATA6), coexpression of functional markers (SFTPB, SFTPC, ABCA3, and AQP5) enables distinction between these two populations in vitro.

**Table 1.** Genes Commonly Expressed in the Lung and Endoderm Lineage

*Gene*	*Function*	*Associated cell type(s)*
*NKX2.1*	Transcription factor	Lung bud/distal airway/thyroid
*FOXP2*	Transcription factor	Lung bud endoderm
*HHEX*	Transcription factor	Thyroid/liver endoderm
*GATA6*	Transcription factor	Lung bud endoderm/distal Lung
*IRX1*	Transcription factor	Lung endoderm
*FOXA2*	Transcription factor	Lung endoderm
*PAX8*	Transcription factor	Thyroid endoderm
*PAX9*	Transcription factor	Thyroid endoderm
*FOXJ1*	Transcription factor	Mature ciliated lung endoderm
*SOX17*	Transcription factor	Mature ciliated lung endoderm
*MUC5AC*	Extracellular glycoprotein	Mature upper airway goblet cells
*SFTPC*	Secreted protein	Mature distal airway type II
*ABCA3*	Membrane bound transporter	Mature distal airway type II
*CFTR*	Transmembrane channel	Mature distal airway type II
*MUC1*	Extracellular glycoprotein	Mature distal airway type II
*SFTPB*	Secreted protein	Mature distal airway type II
*SFTPC*	Secreted protein	Mature distal airway type II
*AQP5*	Transmembrane channel	Mature distal airway type I
*PDPN*	Transmembrane protein	Mature distal airway type I/kidney podocytes
*P2X7*	Receptor	Mature distal airway type I/macrophages/CNS
*PAX6*	Transcription factor	Forebrain
*TG*	Secreted protein	Thyroid
*CK18*	Cytoskeletal protein	Epithelium
*CD26*	Cell surface protein	Lungs/kidneys
*ZO1*	Tight junction protein	Epithelium
*SOX2*	Transcription factor	Oesophagus/pluripotency
*NANOG*	Transcription factor	Pluripotency
*POU5F1*	Transcription factor	Pluripotency
*TRA-1-60*	Cell surface protein	Pluripotency

Several cell subtypes within the lung express unique markers. The most common markers included in this study, along with their function and most commonly associated cell type, are included.

Here, we extend our previous study by showing that fibroblast growth factor (FGF)10 combined with retinoic acid (RA) drives differentiation of hFSCs into cells representative of the lung bud, which can then produce cells with a profile similar to the AECTI and AECTII-like cells of the distal airways. The resulting AEC-like cells display characteristics of their in vivo counterparts such as NKX2.1 and GATA6 gene expression, as well as functional characteristics such as surfactant secretion and CFTR activity. We demonstrate the clinical relevance of our system by using hFSCs derived from patients with cystic fibrosis (CF) to model key aspects of CF-associated lung disease in vitro, including defective chloride transport across the cell membrane. Furthermore, we validate the importance of our cells as a drug screening system by reproducing the effects of VX809, a small-molecule compound restoring CFTR function, currently in phase II clinical trials. Considered together, these results demonstrate the potential of hFSCs as a novel and highly efficient platform for the generation of distal RECs, with diverse clinical applications toward studying lung development, modeling respiratory disease in vitro, and validating novel therapeutic compounds.

## Materials and Methods

### Generation of hIPSCs

hIPSCs (BBHX8, A1TATD) were derived using retrovirus-mediated reprogramming of human skin fibroblasts using the Yamanaka factors. 508CFTR-1, 508CFTR-2 were derived using sendi virus-mediated reprogramming using the Yamanaka factors.

### Maintenance of hESCs and hIPSCs

hESCs (H9) and hIPSCs (BBHX8, A1ATD-1, 508CFTR-1, 508CFTR-2) were cultured in a chemically defined, feeder-free culture system as previously described using Activin-A (10 ng/mL) and basic fibroblast growth factor (bFGF) (12 ng/mL). Cells were passaged every 7 days using collagenase for hESCs or a mixture of dispase and collagenase at a ratio of 1:1 for hIPSCs [6,13].

### Differentiation of hIPSCs and hESCs into endoderm

Pluripotent cells that were either hESCs or hIPSCs were differentiated into definitive endoderm using CDM-PVA and Activin-A (100 ng/mL), bone morphogenetic protein (BMP)4 (10 ng/mL), bFGF (20 ng/mL), and LY294002 (10 μM) for 3 days as previously described [6,13]. For hIPSC differentiation, cells were treated on day 1 of differentiation with CHIR 99021 (10 μM) for 24 h. CHIR 99021 was omitted during day 2 and 3 of endoderm differentiation. A complete protocol has been previously published [6,13].

### Patterning of definitive endoderm

After differentiation into definitive endoderm, cells were cultured in RPMI+B27 medium with Activin-A (50 ng/mL) for 3–4 days to generate foregut cells. Foregut cells could be expanded over time and stably expressed foregut markers, including SOX17, HNF4a, and GATA4 as previously described [6].

### Differentiation of foregut endoderm to lung epithelium

Foregut endoderm was cultured in RPMI medium containing FGF10 (100 ng/mL) and RA (1 μM) for 4–6 days to produce a population of NKX2.1/FOXP2-positive HHEX-negative cells. Cells were then cultured in RPMI medium containing FGF10 (100 ng/mL) and hepatocyte growth factor (HGF, 50 ng/mL) for a further 10 days. Cells were then matured for at least 15 more days in RPMI medium containing FGF10 (100 ng/mL).

### Fetal and adult primary tissue samples

Quantitative polymerase chain reaction (QPCR) was performed using human primary fetal lung (FL) control and an adult distal airway epithelium control. The FL control is representative of both lobes of a 7.5 week fetal sample, while the adult sample is representative of RNA from the lower left lobe of a healthy male donor (AMSBIO). The hIPSCs used in our studies were derived from fibroblasts obtained from the Coriell depository (CF) or obtained from informed patients using the full ethical approval 08/H0331/201. The hESCs were obtained from WiCell and were used under the United Kingdom stem cells steering approval SCSC10-44. Human fetal tissues were obtained under the ethical approval 13/NW/0205.

### RNA isolation, reverse transcription, and QPCR

RNA was isolated using either the GenElute (Sigma-Aldrich) mammalian total RNA isolation kit or RNeasy Kit (Qiagen). Cells were washed with phosphate-buffered saline (PBS) and then lysed in 350 μL of RNA lysis buffer. RNA was purified as per the manufacturer's instructions. DNA digestion was performed using RNAase-free DNase (Sigma) as per the manufacturer's recommendations. Five hundred nanogram of total RNA was reverse transcribed using 500 ng total RNA, 0.5 μL random primers (Promega), and 1 μL of dNTP's (Promega) per reaction. Samples were heated to 65°C for 5 min and then placed on ice for a further 5 min. Four microliter of first-strand buffer (Invitrogen)+2 μL dithiothreitol (Invitrogen)+1 μL RNAse OUT (Invitrogen)+0.5 μL SuperScript II (Invitrogen) was added to each sample and incubated at room temperature for 10 min, followed by 42°C for 75 min and 70°C for 15 min. cDNA was diluted into a total volume of 500 μL of RNAse-free water. Five microliter of cDNA per reaction was combined with 7.5 μL Cyber-Green Sensi mix (Bioline), 0.6 μL each of forward primer and reverse primers, and 1.3 μL of RNAse-free water. PCR was performed using a Stratagene Thermocycler by using one cycle at 95°C for 10 min, then 40 cycles of 95°C for 30 s, 60°C for 30 s, and 72°C for 30 s followed by one cycle at 95°C for 1 min. All QPCR data show an average of three experiments, and error bars represent standard error of the mean. hESCs (H9) were used as a negative control in all the experiments, and the internal reference gene used for all QPCR was hydroxymethylbilane synthase. Statistical analysis was performed using the GraphPad Prism Software version 6, and all statistical significance was calculated using a standard one-way analysis of variance. Levels of significance are represented as *P≤0.05, **P≤0.01, ***P≤0.001, and ****P≤0.0001.

### Immunocytochemistry

Cells were fixed for 20 min at 4°C in 4% paraformaldehyde and washed in PBS. Cells were incubated for 20 min at room temperature in phosphate buffered saline Triton (PBST) (0.1% Triton X-100; Sigma; in PBS) containing 10% donkey serum (Serotec Ltd.) and incubated overnight at 4°C with primary antibody diluted in 1% donkey serum in PBST. Cells were then washed in PBS and incubated with secondary antibodies for 2 h at room temperature. Hoechst 33258 was used to visualize DNA. See Supplementary Table S1 (Supplementary Data are available online at www.liebertpub.com/scd) for a complete list of primary antibodies used in this study. Samples were imaged using a Zeiss Imager M.2, equipped with AxioCam MRm and MRc cameras and AxioVision software for image capture and scale bars=100 μM.

### Flow cytometry

For a complete list of primary and secondary antibodies used for flow cytometry, please refer Supplementary Table S1. Adherent cells were washed twice in PBS and then incubated for 20 min at 37°C in cell dissociation buffer (Invitrogen). Cells were dissociated by gentle pipetting and resuspended at ∼0.1–1×10^6^ cells/mL in PBS. For live cell surface staining, cells were blocked for 30 min using 10% donkey serum before staining. For internal markers, cells were pelleted and fixed by resuspending cells in 4% paraformaldehyde solution at 4°C for 20 min. Cells were washed in PBS, then blocked, and permeabilized in PBS containing 10% donkey serum and 0.01% Triton X-100. Cells were incubated in 1% donkey serum 0.001% Triton X-100 containing the primary antibody overnight at 4°C. Cells were then washed in PBS 1% donkey serum and incubated with secondary antibodies overnight at 4°C. Cells were analyzed using an FACS Calibur machine (BD Biosciences). All flow cytometry experiments were gated first using unstained cells and then cells containing the secondary antibody only. On all flow cytometry plots, the secondary-only population is shown in gray. All gates shown on scatter and histogram plots were set to the secondary only control. All flow cytometry was validated with immunocytchemistry to ensure false-positive or -negative results were not recorded.

### CFTR assay

Lung epithelial cells were transferred to 35 mm glass-bottom tissue culture dishes 5 days before the CFTR assay. Wild-type and CFTR mutant hPSCs were treated with or without the small molecules VX809 for 48 h (30 mM) before the commencement of the assay or transferred to an incubator at 28°C 6 h before the beginning of the assay. Cells were then loaded with the chloride-sensitive dye [N-(ethoxycarbonylmethyl)-6-methoxyquinolinium bromine] (MQAE) (Life Technologies) for 4 h before performing the assay. Ringer's solution was made to measure chloride influx. Standard Ringers solution consisting of 140 mM NaCl, 5 mM KCl, 1.5 mM CaCl, 1 mM MgCl, 10 mM glucose, and 4 mM HEPES was used as the chloride positive solution. To measure chloride efflux, Ringers solution was made as described earlier by exchanging Cl for NO_3_, 140 mM NaNO_3_, 5 mM KNO_3_, 1.5 mM CaNO_3_, 1 mM MgNO_3_, 10 mM glucose, and 4 mM HEPES. A perfusion insert was then placed inside the 35 mm organ culture plates containing lung epithelial cells loaded with MQAE (8 mM). A vacuum pump was attached to the outlet manifold, and Ringers solution was injected through the inlet port. Images were captured on an inverted microscope measuring fluorescent acitivity at an excitation wavelength of 370 nm and an emission at 450 nm. Live images were captured at a rate of four frames per second, and different solutions were added. A CFTR inhibitor (CFTR-172; Sigma; 10 μM) was added to demonstrate specificity to the CFTR. Fluorescent intensity of at least 10 cells per frame was measured using ImageJ Software. MQAE florescence is inhibited in the presence of chloride ions via diffusion-limited collisional quenching. In chloride-rich medium, net flow of chloride ions will be into the cell quenching MQAE signal; in chloride-deficient medium (NO_3_^−^), net flow of chloride ions is out of the cell, causing an increase in MQAE fluorescence.

### Three-dimensional lung culture

To produce branching cell clusters, NKX2.1/FOXP2 cells were dissociated using cell dissociation buffer and transferred to cold Matrigel. Matrigel was prepared according to the manufacturer's recommendations and supplemented with FGF10 (100 ng/mL). Dissociated cells were washed and then mixed with Matrigel. Fifty microliter of matrigel and cells were then blotted onto wells of a pre-warmed 24-well plate to produce solid spheres of Matrigel and cells. Medium containing FGF10 was then added. To produce cystic distal airway three-dimensional (3D) cultures, NKX2.1/SFTPC-positive cells were dissociated and mounted in Matrigel containing FGF10 as described earlier.

## Results

### FGF10 and RA generate NKX2.1^+^, FOXP2^+^, and HHEX^−^ lung progenitor cells

Our overall objective was to develop a methodology for differentiation of hFSCs into RECs. To achieve this, we first focused on the differentiation of hFSCs into cells corresponding to the lung bud. We have previously demonstrated the capacity of hFSCs to spontaneously differentiate into lung and/or thyroid progenitors [6]. Indeed, when injected under the kidney capsule of immunodeficient mice, hFSCs form cystic structures containing cells expressing the lung/thyroid marker NKX2.1 [6]. However, little is known about the signaling cascades controlling lung specification in the foregut. FGF10 is considered the key pathway involved [14,15]. RA is also implicated in early lung development, as disruption of RA signaling in mice leads to poorly developed lungs and the use of RA receptor antagonists prevents foregut explant cultures from developing the lung primordium [16].

**FIG. 1.**
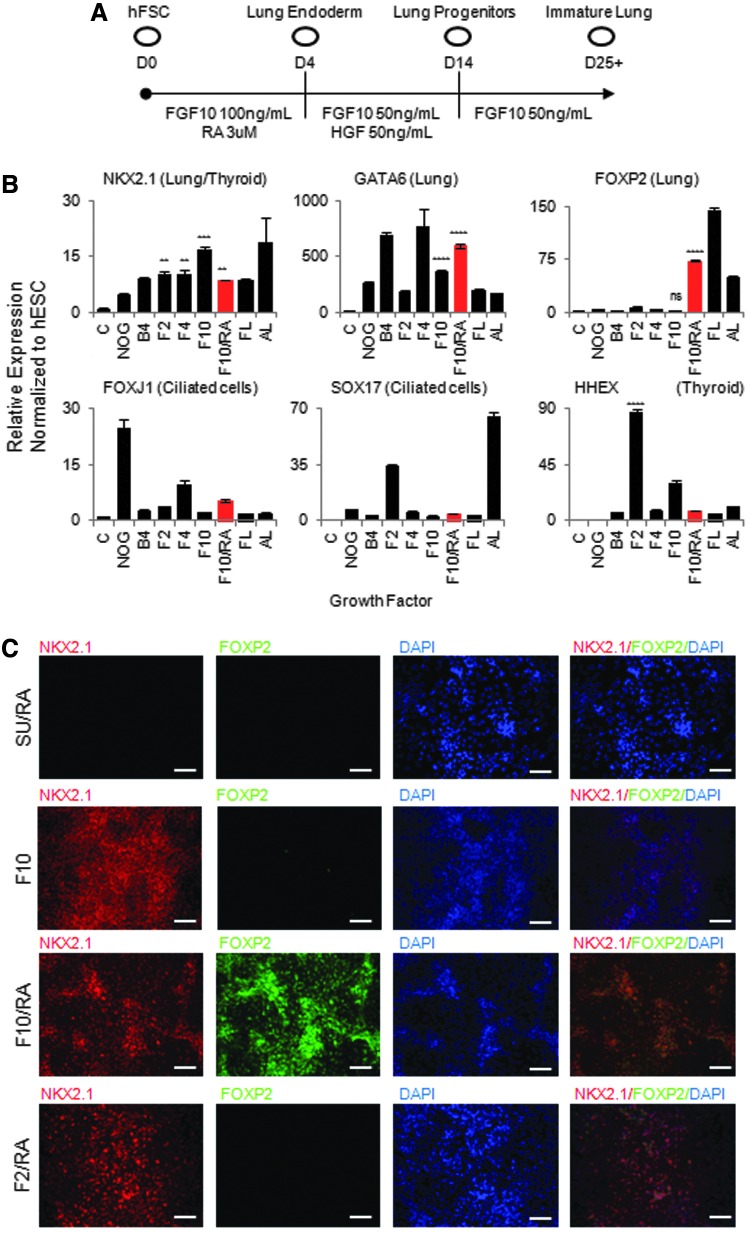
RA and FGF10 pattern foregut endoderm into NKX2.1/FOXP2-positive lung endoderm. **(A)** Protocol to produce lung endoderm progenitors and distal lung epithelium. **(B)** Quantitative polymerase chain reaction (QPCR) analysis showing hFSCs cultured in medium containing FGF10 with or without additional growth factors express early lung progenitor genes (NKX2.1, FOXP2, and GATA6) and do not express more mature lung genes (SOX17, FOXJ1) or genes associated with thyroid progenitors (HHEX). **(C)** Immunocytochemistry showing hFSCs cultured in medium containing FGF10 and RA coexpress early lung progenitor genes (NKX2.1, FOXP2), while hFSCs cultured in the presence of FGF inhibitor SU5402, FGF10 without RA, or FGF2 do not express these genes. *White bars*=100 μM. *P≤0.05, **P≤0.01, ***P≤0.001, ****P≤0.0001. RA, retinoic acid; FGF, fibroblast growth factor; hFSCs, human foregut stem cells; FL, human fetal lung control; AL, adult lung control; C, undifferentiated hESC control; hESC, human embryonic stem cell; ns, not significant.

To generate early lung progenitors in vitro from hFSCs, we decided to study the effect of multiple growth factors known to direct lung specification, including FGF10 and RA [14,15,17]. We first treated hFSCs with a combination of FGF2, FGF4, and FGF10 (Fig. 1A, B) and found that all three FGFs are sufficient to induce expression of lung bud markers NKX2.1, GATA6, IRX1, and FOXA2 at levels similar to those of human FL (Fig. 1B and data not shown). However, in contrast to FGF10, FGF2 induced HHEX and PAX8 mRNA expression along with NKX2.1, suggesting that this pathway could promote hFSCs differentiation into a thyroid-like cell population (Fig. 1B, C; Supplementary Fig. S1A). Analyses of other signaling pathways such as transforming growth factor (TGF)β revealed that they did not play a significant role in inducing the pulmonary endoderm phenotype (Supplementary Fig. S1A, B). Considered together, these results demonstrate that FGF signaling is sufficient and necessary for specification of hFSCs into lung and thyroid progenitors with FGF2 inducing thyroid markers and FGF10 more specific for lung markers.

Although all FGFs were able to induce NKX2.1, none were able to induce coexpression of the lung bud marker FOXP2. To determine whether multiple signal pathways were required to specify the lung domain in vitro, we decided to characterize the effect of combinations of growths factors, including members of the FGF, TGF, and Wnt pathways. RA was the only factor identified by this screen to have a synergistic effect with FGF10. Indeed, supplementation of FGF10 with RA increased cell proliferation (data not shown), upregulated the lung bud marker FOXP2 to levels observed in FL (Fig. 1B), and suppressed the expression of the thyroid markers HHEX, PAX8, and PAX9 (Fig. 1B; Supplementary Fig. S1A). Coexpression of NKX2.1 and FOXP2 was confirmed by immunostaining (Fig. 1C), while the expression of mature lung markers such as FOXJ1, SOX17, MUC5AC, and SFTPC (Fig. 1B; Supplementary Fig. S1A) was not detected, confirming that the resulting cells correspond to early lung progenitor cells, rather than mature RECs. Importantly, these progenitor cells could not be generated in the presence of FGF10 or RA alone, as well as RA combined with FGF2 (Fig. 1C). Collectively, these results demonstrate that a combination of FGF10 and RA is required to produce pulmonary endoderm in vitro corresponding to the lung bud.

### NKX2.1^+^/FOXP2^+^ lung progenitors produce distal airway epithelium similar to AECTII and AECTI cells

**FIG. 2.**
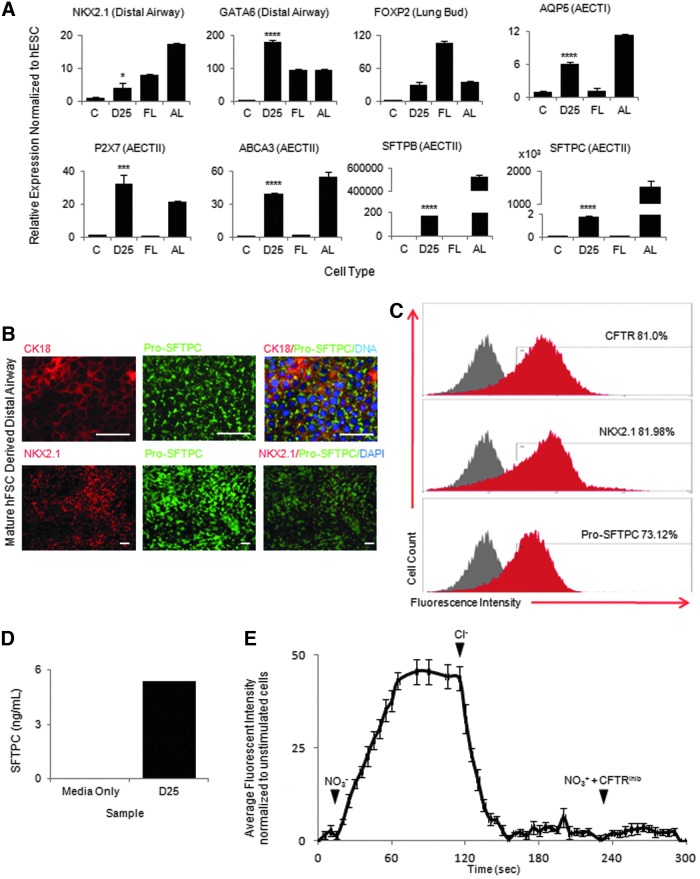
NKX2.1/FOXP2-positive lung progenitors mature into distal AEC. **(A)** QPCR analysis showing NKX2.1^+^/FOXP2^+^-positive lung progenitor cells cultured for a further 25 days downregulate lung bud markers (FOXP2) and express genes of the distal airway epithelium (NKX2.1, GATA6), AECTII (SFTPC, SFTPB, ABCA3, and P2X7), and AECTI (AQP5). **(B)** Immunocytochemistry showing matured lung endoderm coexpressing distal airway markers (Pro-SFPTC, CK18, and NKX2.1). **(C)** Flow cytometric analysis showing percentages of cells positive for distal airways markers (CFTR, NKX2.1, and Pro-SFTPC). *Red* shading=positive stained *Gray* shading=isotype/secondary control. **(D)** SFTPC enzyme-linked immunosorbent assay detection of SFTPC in tissue culture medium from cultures of matured distal airway epithelium. **(E)** Fluorescent trace of Cl^−^ indicator dye (MQAE) showing CFTR activity in matured airway epithelium. No fluorescent signal was detected within lung epithelium using Cl^−^ containing medium (*t*=0 s), CFTR activity was shown by an increase in cell fluorescence in medium containing NO_3_^−^ (*t*=15 s) and then a loss of fluorescence when Cl^−^ medium was added back to cells (*t*=115 s). Cells treated with NO_3_^−^ medium and a CFTR inhibitor showed no increase in fluorescence inside lung epithelial cells (*t*=230 s). *White bars*=100 μM. *P≤0.05, ***P≤0.001, ****P≤0.0001. AEC, alveolar epithelial cell; CFTR, cystic fibrosis transmembrane conductance regulator; FL, human foetal lung control; AL, adult lung control; C, undifferentiated hESC control; D25, airway epithelium cultured for 25 days; MQAE, N-(ethoxycarbonylmethyl)-6-methoxyquinolinium bromine.

We next decided to characterize the capacity of the NKX2.1^+^/FOXP2^+^ lung progenitors to mature into a more adult-like airway epithelium. In the mouse, the lung bud branches and invades the surrounding mesenchyme in a process largely driven by FGF10 from the surrounding mesenchyme. The production of distal airway epithelial cells that occurs gradually over the canalicular and saccular stages of FL development is also dependant on a continuous FGF signal. We therefore decided to test the effect of prolonged FGF10 signaling on the NKX2.1/FOXP2-positive population. Cells cultured in medium containing FGF10 and HGF for 10 days and then FGF10 alone for a further 15 days continued to express markers indicative of the distal tip airway epithelium (*NKX2.1* and *GATA6*) and downregulated the lung bud markers (*FOXP2*) (Fig. 2A), suggesting that cells were adopting a more distal REC fate. Indeed, FGF10 exposure induced expression of several mature lung epithelium markers, predominantly characteristic of AECTII (*ABCA3*, *CFTR*, *MUC1*, *SFTPB*, and *SFTPC*) and AECTI (*AQP5*, *PDPN* and *P2X7*) (Fig. 2A and data not shown) in the absence of markers of other lineages such as the forebrain (*PAX6*) or the thyroid (*HHEX*, *PAX8*, *PAX9*, and *TG*; Supplementary Fig. S2A). Coexpression of NKX2.1 and the AECTII marker Pro-SFTPC; Pro-SFTPC and the epithelial marker CK18; as well as a cell surface marker CD26 and CFTR was further confirmed by immunostaining (Fig. 2B; Supplementary Fig. S2B). Of note, CFTR is expressed in a variety of endodermal cell types, including cells of the intestines and pancreas; however, co-immunocytochemistry of the CFTR with PDX1 (Pancreas) or CDX2 (intestine) revealed cells that were negative for these lineage markers and instead expressed high levels of the distal airway marker NKX2.1 (Supplementary Fig. S2D). Flow cytometric analyses revealed that 80% of cells expressed NKX2.1 and CFTR, and 70% expressed Pro-SFTPC (Fig. 2C). Importantly, the resulting cells did not exhibit significant expression of secretory cell markers (*MUC5AC*) or markers of ciliated cells (FOXJ1, SOX17), suggesting that FGF10 supports development of more distal lineages rather than proximal airway cell types (Supplementary Fig. S2A).

In vivo, distal AECs are organized in 3D alveolar structures, exhibiting apico-basal polarity. The capacity of our system for the generation-polarized epithelial cells in two-dimensional culture was demonstrated by confocal microscopy, which confirmed apical localization of CFTR and CD26 and lateral localization of the tight junction marker ZO1 as expected (Supplementary Fig. S2B, C). Finally, 3D culture resulted in the formation of lung organoids that retained both their gene expression profile and functional characteristics, demonstrating the potential of our platform as a system for studying 3D lung morphogenesis and disorders affecting distal airway formation (Supplementary Fig. S3A–E). Considered collectively, these results underline the capacity of NKX2.1/FOXP2-positive cells for differentiation into distal AECTII, AECTI, and AEC organoids under continued FGF10 stimulation.

### hFSCs-derived AECs show airway functionality in-vitro

The airway epithelium we generated expresses two important functional genes, surfactant protein C (SFPTC) and the ABCA3 protein, a major component of the lamellar bodies that allow secretion of surfactant proteins. To determine whether hFSCs-derived AECTII were indeed functional, we performed an enzyme-linked immunosorbent assay for SFPTC and found that after 24 h of culture SFTPC could be detected in the tissue culture medium (Fig. 2D). To assess the functionality of CFTR usually expressed in AECTII and AECTI cells, cells were loaded with a chloride-sensitive indicator dye (MQAE). Chloride transport across the cell membrane in AECs relies largely on CFTR. Fluorescence microscopy revealed that intracellular chloride levels increased in response to a chloride-rich medium and decreased in response to a chloride-deficient medium, thereby confirming CFTR functionality. Importantly, using a CFTR inhibitor, we demonstrated that this chloride influx and efflux was indeed CFTR dependant (Fig. 2E). Considered together, these observations illustrate that distal AECs derived from hFSCs recapitulate key functional characteristics of their in vivo counterparts.

### hIPSCs from CFTR mutant fibroblasts form distal AECs

Despite significant progress modeling diseases in other organs [18,19], few stem cell platforms are specifically aimed at disease modeling in the lung. To validate the clinical application of our culture system, we decided to model CF, one of the most common genetic disorders in the world, affecting as many as 1:2,000 births in Europe, with lung transplantation remaining the only effective treatment [20–22]. CF is caused by mutations in the CFTR, which result in deregulation of chloride and water transport across the lung epithelium, manifesting as an increase in airway mucus viscosity. This causes poor mucus and pathogen clearance, leading to inflammatory changes in the lung parenchyma, fibrosis, and reduced pulmonary function [23].

**FIG. 3.**
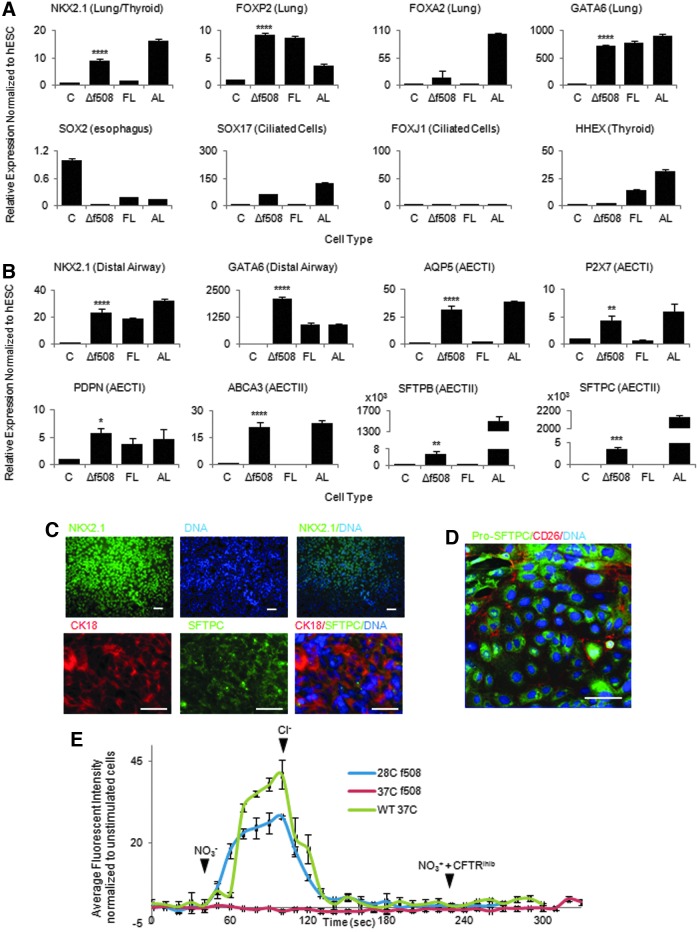
CFTR-Δf508-derived hFSCs can differentiate into lung epithelium. **(A)** QPCR analysis showing that hFSCs derived from patients with the CFTR mutation Δf508 can differentiate into early lung endoderm and express early markers (NKX2.1, FOXP2, GATA6, and FOXA2) while they are negative for more mature markers (FOXJ1, SOX2, and SOX17) and thyroid markers (HHEX). **(B)** QPCR analysis showing that CFTR mutant lung progenitors can develop into mature airway epithelium and continue to express distal airway markers (NKX2.1, GATA6) as well as AECTII markers (SFTPB, SFTPC, and ABCA3) and AECTI markers (AQP5, P2X7, and PDPN). **(C)** Immunocytochemistry showing distal airway epithelium expresses NKX2.1, SFTPC, and CK18. **(D)** Confocal microscopy showing localization of Pro-SFTPC in the cytoplasm of the distal airway epithelium. **(E)** Trace of chloride influx and efflux in mature airway epithelium from CFTR-human-induced pluripotent stem cell showing the temperature sensitivity of CFTR. Chloride influx and efflux was measured in mature airway epithelium expressing the wild-type CFTR (*green* trace) cultured at 37°C, the Δf508 mutation (*red* trace) cultured at 37°C, and the Δf508 mutation (*blue* trace) cultured at 28°C. Addition of Cl^−^ or NO_3_^−^ indicated with solid *arrowheads*. *White bars*=100 μM. *P≤0.05, **P≤0.01, ***P≤0.001, ****P≤0.0001. FL, human fetal lung control; AL, adult lung control; C, undifferentiated hESC control; Δf508, airway epithelium from cystic fibrosis patients cultured for 25 days.

The Δf508 constitutes the most common mutation in CF. Skin fibroblasts obtained from a patient carrying the CFTR Δf508 mutation were reprogrammed into hIPSCs using non-integrative Sendai virus expressing the four factors Oct-4, Sox2, C-Myc, and KLF-4 [24]. The resulting CF-hIPSC lines were characterized for their pluripotent state, capacity of differentiation into the three germ layers, and absence of transgene integration as previously described (Supplementary Fig. S4A and data not shown) [19]. CF-hIPSCs were then differentiated into distal AECs as described earlier (Fig. 3A–D; Supplementary Fig. S4B, C), and the functionality of the CFTR protein in the resulting cells was tested using the MQAE assay. As expected, CF-derived lung cells did not show a change in fluorescence, indicating their CFTR was not functional (Fig. 3E). Interestingly, the Δf508 mutation causes a temperature-sensitive mis-folding of the CFTR whereby the activity of the Δf508-CFTR has been shown to be normal at ∼28°C with mis-folding at 37°C [25–27]. Accordingly, the MQAE assay showed a change in fluorescence in CF-hFSCs-derived AECs grown at 28°C for 6 h before the MQAE assay, demonstrating that the functionality of CFTR could be restored by a reduction in temperature (Fig. 3E). Taken together, these data demonstrate that our culture system enables the production of lung epithelial cells that recapitulate the basic biology of the Δf508 mutation in vitro.

**FIG. 4.**
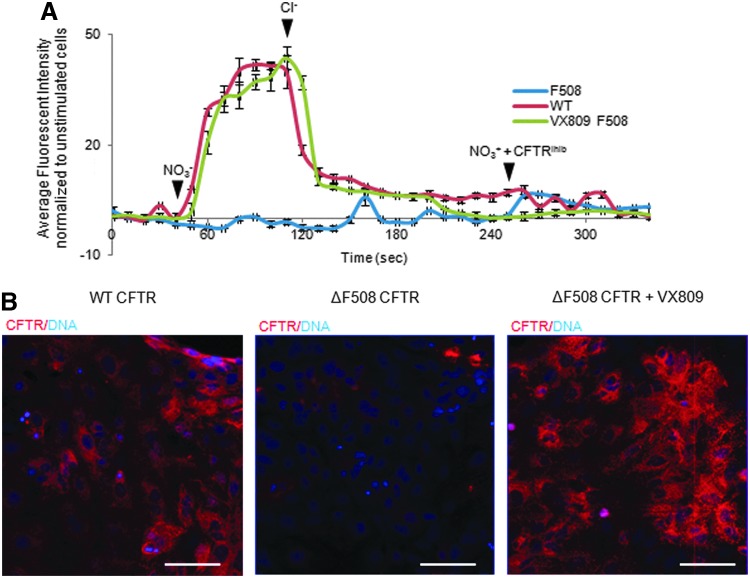
The CFTR-Δf508 mis-folded receptor can be rescued to a functional state using the small molecule VX809. **(A)** Chloride influx and efflux trace in mature airway epithelial cells expressing the wild-type CFTR (*red* trace), the ΔF508 mutation in the CFTR (*blue* trace), or the ΔF508 mutation in the CFTR but treated with the small molecule VX809 (*green* trace). **(B)** Confocal microscopy showing expression of CFTR in matured airway epithelium from wild-type cells, cells with the ΔF508 mutation in the CFTR, and cells with the ΔF508 mutation in the CFTR but treated with the small molecule VX809. *White bars*=100 μM. Addition of Cl^−^ or NO_3_^−^ indicated with solid *arrowheads*.

Finally, to characterize the potential of our system as a platform for validating novel therapeutic agents, we decided to assess the efficiency of the small molecule VX809 for restoring CFTR function in CF-hFSCs-derived AECs. Indeed, VX809 has been shown in several studies to correct folding defects in the mature CFTR protein allowing reduced degradation, increased trafficking to the cell surface, and restoration of chloride transport. CF-hFSCs-derived lung cells were incubated for 48 h with or without VX809 and then loaded with MQAE for detection of chloride transport. CF-hFSCs-derived AECs grown in the presence of VX809 showed a dramatic improvement in the capacity to transport chloride, confirming the efficiency of this drug to correct CF in vitro (Fig. 4A). Furthermore, confocal microscopy revealed that CFTR was able to accumulate to greater levels on VX809 treatment (Fig. 4B). Taken together, these data confirm the mode of action of VX809 and thus demonstrate that hFSCs-derived distal AECs could be used to predict the activity of small molecules to correct lung diseases in vitro.

## Discussion

Here, we describe the development of a novel platform for the differentiation of hFSCs into functional distal AECs recapitulating key stages of lung development and provide a new system to model pulmonary diseases in vitro. Our observations expand on previous studies illustrating the differentiation capacity of hFSC toward liver and pancreatic lineages and underline their potential for generating cell types from the most anterior part of the foregut such as the thyroid and lung.

Remarkably, our protocol allows the production of a population of lung cells expressing mainly AECTII and AECTI markers (80% NKX2.1/CFTR/Pro-SFTPC). This bias toward distal lung cells is not specific to hFSCs since previous reports describe similar results with direct differentiation of hPSCs when using a combination of RA and FGF10 [3,4]. The use of BMP4 at an early stage of differentiation could favor proximal lung cell production [4]; however, further studies are required to understand the mechanisms involved in the proximo-distal patterning of the lung progenitors in vitro and hFSCs could facilitate such analyses, as they represent the closest embryonic stage to lung bud induction in humans.

Indeed, we have already used hFSCs to identify key signaling pathways controlling pulmonary differentiation. Importantly, the function of FGF10 and RA in inducing lung progenitor differentiation is in agreement with in vivo studies [11,14,28] and this similarity reinforces the application of hFSCs as a model to study early stages of lung specification.

Importantly, the use of hFSCs versus hPSCs provides significant advantages in terms of clinical applications. The clinical significance of hFSCs-derived RECs was demonstrated by modeling CF in vitro and by validating the effects of VX809 on rescuing the disease phenotype. Similar results have been reported with existing platforms [4]; however, in contrast to hPSC-based systems, hFSCs-derived AECs can be produced from any hIPSC line, even those resistant to endoderm differentiation [6]. This improvement could facilitate the generation of large cohorts of patient-specific AECs, which are required for the study of polygenic or multifactorial disorders in vitro. Furthermore, hFSCs limit variability between experiments since they bypass the need for endoderm differentiation, which can be extremely variable between experiments and different hIPSC lines. Similarly, the generation of AECs from hFSCs is more time and cost effective when compared with direct differentiation of hPSCs and, thus, represents a significant benefit for large-scale applications such as drug screening.

In conclusion, our results demonstrate that hFSCs-derived AECs can be used to study human early lung development, drug screening, and disease modeling. More importantly, this system represents an important technological advance that is capable of bridging the gap between isolated proof-of-principle studies and the large-scale clinical applications required to fully exploit the translational potential of stem-cell-derived AECs.

## Supplementary Material

Supplemental data

Supplemental data

Supplemental data

Supplemental data

Supplemental data
