# Supplementary material for: Generation of Distal Airway Epithelium from Multipotent Human Foregut Stem Cells
Source: Stem Cells Dev. 2015 Mar 10;24(14):1680–90. doi: 10.1089/scd.2014.0512 (PMC4499787; doi:10.1089/scd.2014.0512)
Supplement: Supplemental data [file Supp_Table1.pdf]

Supplementary Table S1. List of Primary Antibodies

Epitope	*Supplier*	*Catalogue number*
NKX2.1	AbCam	Ab76013
NKX2.1	AbCam	Ab72876
NKX2.1	SantaCruz	SC13040
FOXP2	R&D	AF5647
HOECHST	SIGMA	B2883
Pro-SFTPC	AbCam	Ab40879
Cytokeratin 18	Santa Cruz	SC6259
CFTR	Santa Cruz	SC10747
CD26	AbCam	Ab3154
E-Cadherin	Millipore	CC43
GATA6	R&D	AF1700
MUC1	AbCam	Ab15481
NANOG	R&D	AF1997
POU5f1	Santa Cruz	SC8628x
TRA-1-60	Santa Cruz	SC21705
SOX2	R&D	AF2018
ZO1	AbCam	Ab59720
