# Supplementary material for: Generation of Distal Airway Epithelium from Multipotent Human Foregut Stem Cells
Source: Stem Cells Dev. 2015 Mar 10;24(14):1680–90. doi: 10.1089/scd.2014.0512 (PMC4499787; doi:10.1089/scd.2014.0512)
Supplement: Supplemental data [file Supp_Fig1.pdf]

Supplementary Data

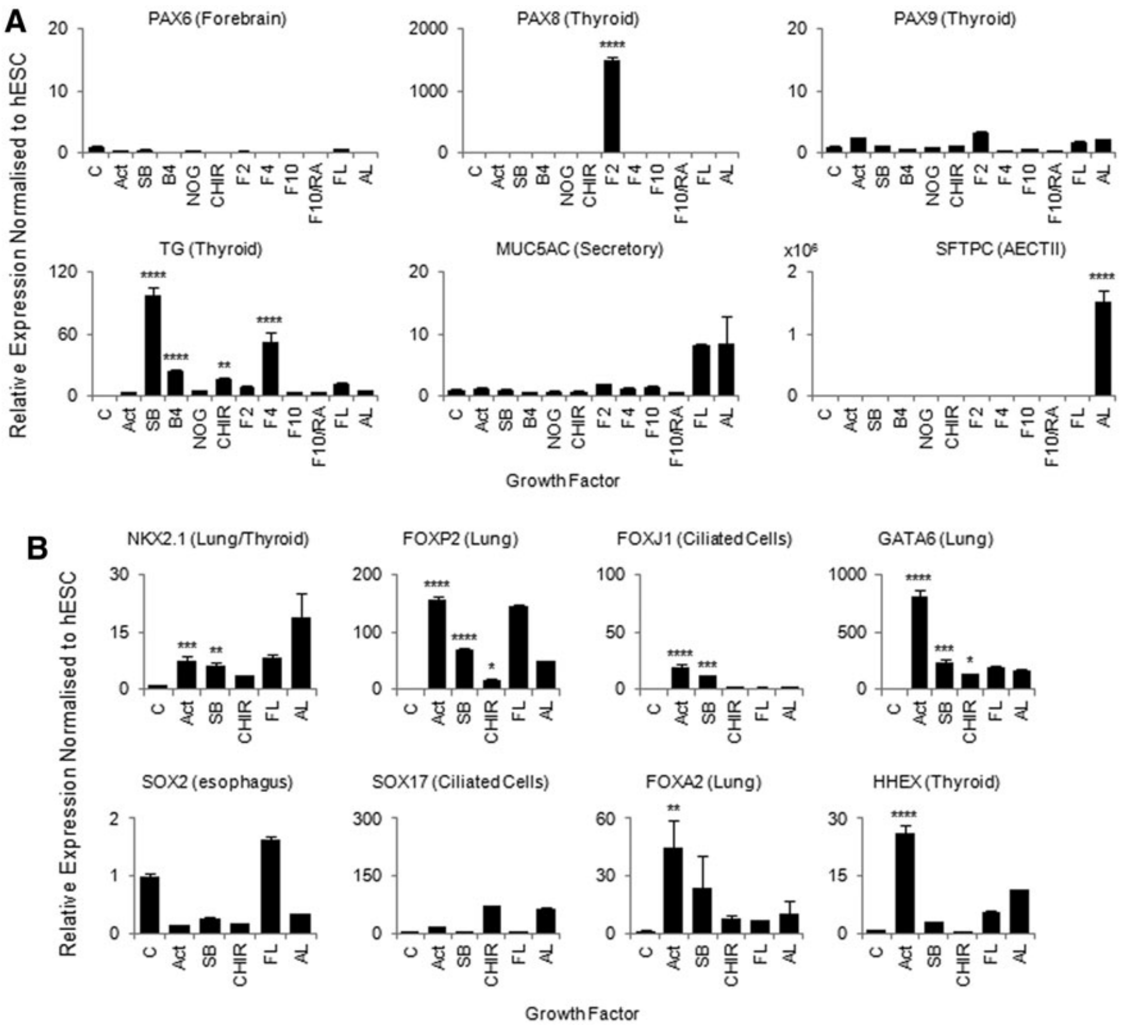

SUPPLEMENTARY FIG. S1. The effect of multiple growth factors on hFSCs to produce lung endoderm. **(A)** Quantitative polymerase chain reaction (QPCR) analysis of early lung progenitor cells showing effects of multiple growth factors on genes involved in thyroid development (PAX8, PAX9, and TG), the forebrain (PAX6), and the mature airway epithelium (MUC5AC, SFTPC). **(B)** QPCR analysis showing pathways Activin, bone morphogenetic protein, and Wnt induce several early lung endoderm genes (NKX2.1, FOXP2, GATA6, and FOXA2), as well as genes associated with more mature lung epithelium (FOXJ1, SOX17, and SOX2) and thyroid specification (HHEX). $*P \leq 0.05$, $**P \leq 0.01$, $***P \leq 0.001$, $****P \leq 0.0001$. hFSC, human foregut stem cells; hESC, human embryonic stem cell; FL, human fetal lung control; AL, adult lung control; C, undifferentiated hESC control.
