# Supplementary material for: Generation of Distal Airway Epithelium from Multipotent Human Foregut Stem Cells
Source: Stem Cells Dev. 2015 Mar 10;24(14):1680–90. doi: 10.1089/scd.2014.0512 (PMC4499787; doi:10.1089/scd.2014.0512)
Supplement: Supplemental data [file Supp_Fig2.pdf]

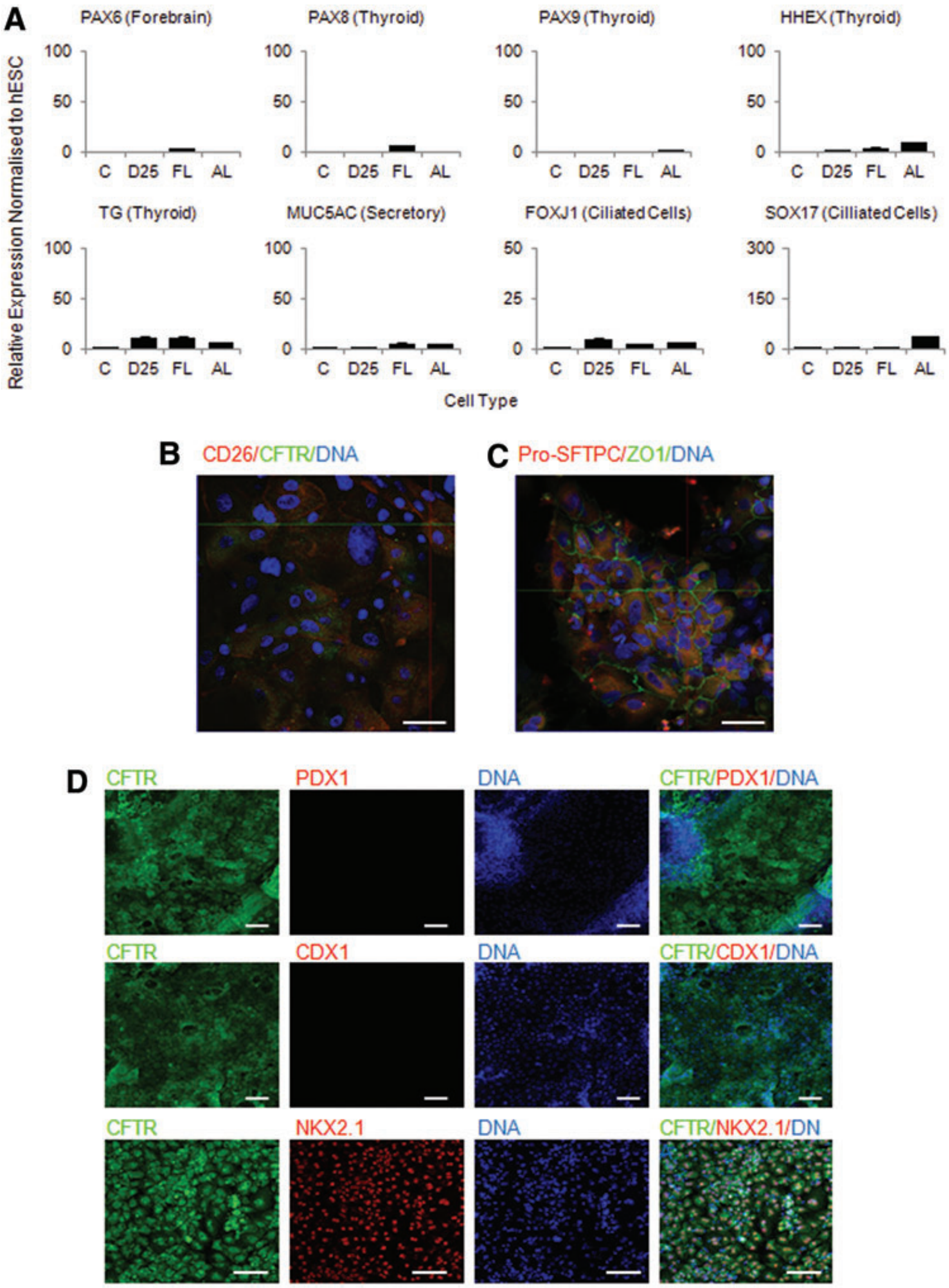

SUPPLEMENTARY FIG. S2. Lung epithelium expresses markers distal lung epithelium and not proximal lineages. **(A)** Matured lung epithelial cells remain negative for markers of the thyroid (PAX8. PAX9, HHEX, and TG), the forebrain (PAX6), and the upper airway epithelium (MUC5AC, FOXJ1, and SOX17). **(B)** Confocal microscopy of polarized mature distal airway epithelium expressing CD26 and CFTR **(C)** or ZO1 and Pro-SFTPC. **(D)** Matured lung epithelium expresses CFTR that colocalizes with NKX2.1 and not markers of the intestine (CDX2) or pancreas (PDX1). *White bars* = 100 μM. CFTR, cystic fibrosis transmembrane conductance regulator; FL, human fetal lung control; AL, adult lung control; C, undifferentiated hESC control; D25, airway epithelium cultured for 25 days.
