# Supplementary material for: Generation of Distal Airway Epithelium from Multipotent Human Foregut Stem Cells
Source: Stem Cells Dev. 2015 Mar 10;24(14):1680–90. doi: 10.1089/scd.2014.0512 (PMC4499787; doi:10.1089/scd.2014.0512)
Supplement: Supplemental data [file Supp_Fig3.pdf]

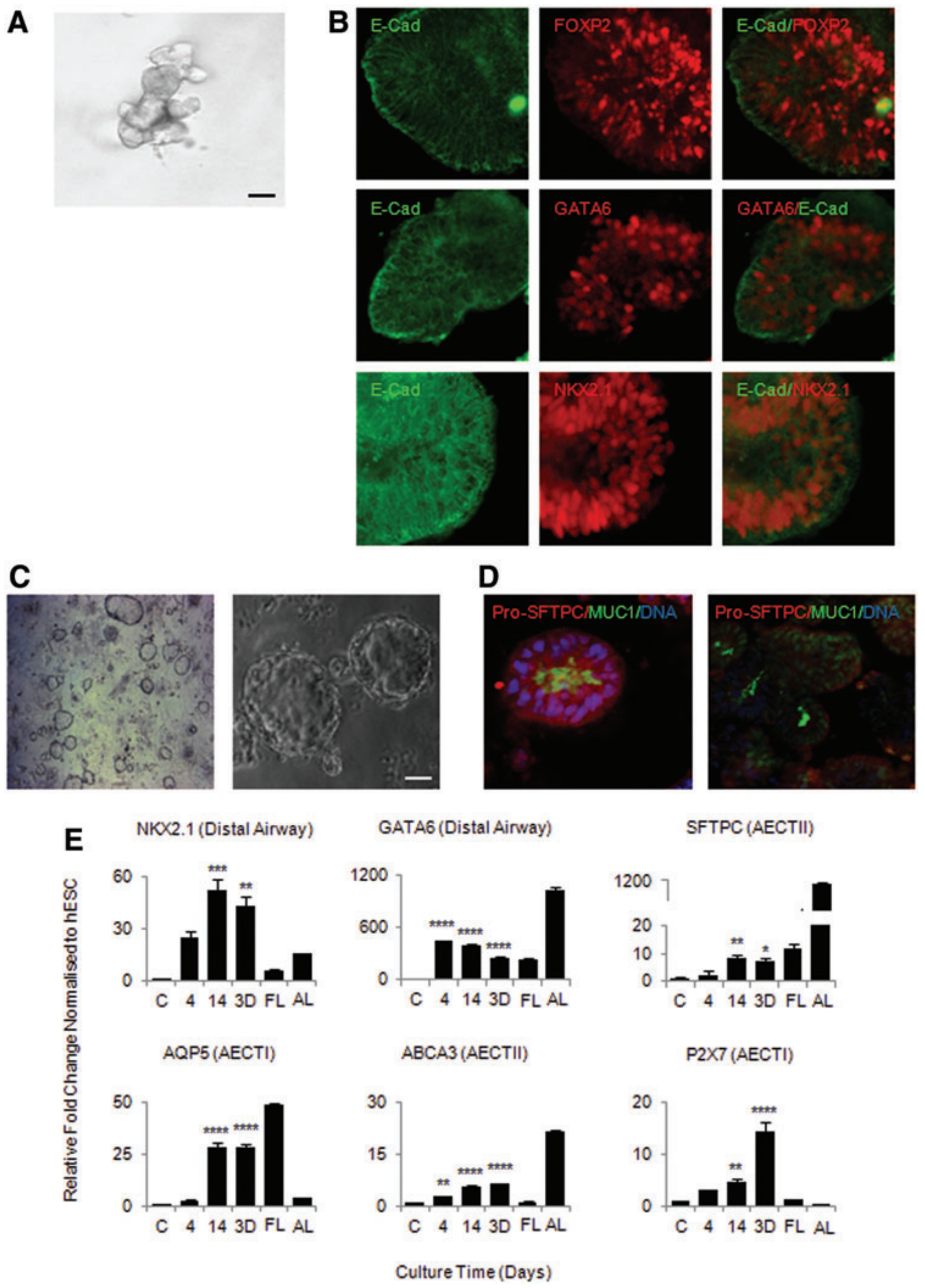

SUPPLEMENTARY FIG. S3. Matured lung epithelium can be cultured in 3D and retains a lung epithelial phenotype. **(A)** NKX2.1/FOXP2 cells form branching clusters of cells when cultured in 3D. **(B)** Immuncytochemistry of branching cells lung endoderm expressing E-cadherin, FOXP2, GATA6, and NKX2.1. **(C)** Distal airway cells cultured in 3D form cystic structure. **(D)** Immunocytochemistry showing 3D cysts continue to express distal airway markers Pro-SFTPC and MUC1. **(E)** QPCR analysis showing that 3D cysts continue to express distal airway markers (NKX2.1, GATA6) as well as genes of AECTI (AQP5, P2X7) and AECTII (ABCA3, SFTPC). *White bars* = 100 μM. *$P \leq 0.05$, **$P \leq 0.01$, ***$P \leq 0.001$, ****$P \leq 0.0001$. 3D, three-dimensional; FL, human fetal lung control; AL, adult lung control; C, undifferentiated hESC control; 3D, airway epithelium cultured for 25 days in 3D Matrigel.
