# Supplementary material for: Generation of Distal Airway Epithelium from Multipotent Human Foregut Stem Cells
Source: Stem Cells Dev. 2015 Mar 10;24(14):1680–90. doi: 10.1089/scd.2014.0512 (PMC4499787; doi:10.1089/scd.2014.0512)
Supplement: Supplemental data [file Supp_Fig4.pdf]

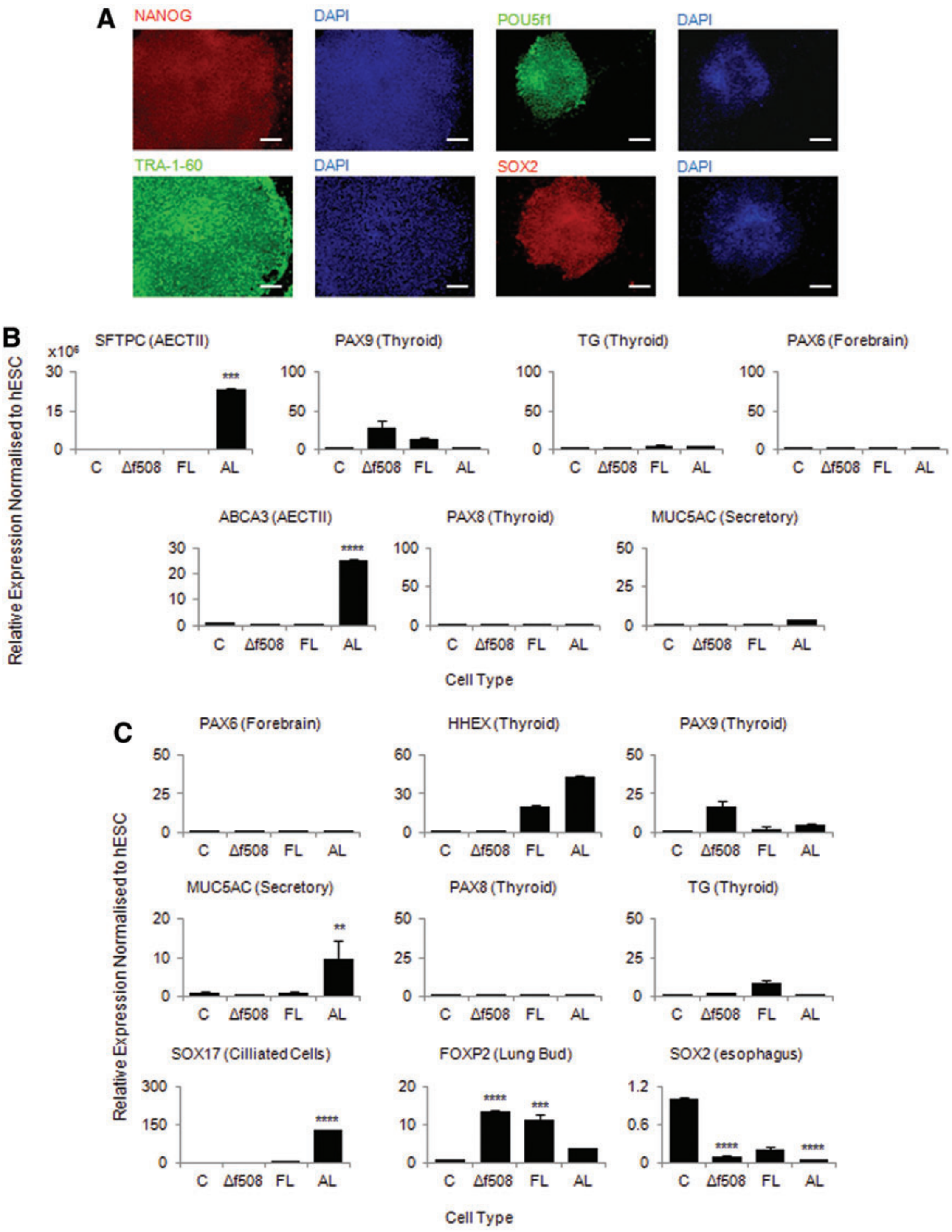

SUPPLEMENTARY FIG. S4. Fibroblasts from CF patients with the ΔF508 mutation make a mature distal lung epithelium. **(A)** Immuncytochemistry showing fibroblasts from ΔF508 patients reprogrammed and express pluripotency genes (NANOG, POU5F1, SOX2, and TRA-1-60). **(B)** ΔF508 lung endoderm does not express markers of the thyroid lineage (PAX9, TG, and PAX8), the forebrain (PAX6), or the more mature airway epithelium (SFTPC, ABCA3, and MUC5AC). **(C)** Mature distal airway epithelium expressing the ΔF508 CFTR continues to express distal airway epithelial genes while remaining negative for thyroid (PAX8, PAX9, and TG), forebrain markers (PAX6), and proximal airway lineages (FOXJ1, SOX2, and SOX17). *White bars* = 100 μM. **$P \leq 0.01$, ***$P \leq 0.001$, ****$P \leq 0.0001$. FL, human foetal lung control; AL, adult lung control; C, undifferentiated hESC control; Δf508, airway epithelium from CF patients cultured for 25 days; CF, cystic fibrosis.
